# A community-based Daoyin program for health promotion: effects of the Qi and mind harmonizing method on body constitution for the health of older adults

**DOI:** 10.3389/fpubh.2025.1644273

**Published:** 2026-01-05

**Authors:** Yun-Ning Tsai, Yu-Hsin Chang, Yi-Chang Su, Shen-Ming Lee, Cheng-Huan Hsiao, Chi-Kuei Lin, Sunny Jui-Shan Lin

**Affiliations:** 1 Department of Chinese Medicine, Tri-Service General Hospital, National Defense Medical University, Taipei, Taiwan; 2 ChanDer Clinic, Taipei, Taiwan; 3 Graduate Institute of Medical Sciences, College of Medicine, National Defense Medical University, Taipei, Taiwan; 4 Department of Statistics, Feng Chia University, Taichung, Taiwan; 5 National Research Institute of Chinese Medicine, Ministry of Health and Welfare, Taipei, Taiwan; 6 E-Med Biotech Inc., Taichung, Taiwan; 7 Department of Chinese Medicine, Shin Kong Wu Ho-Su Memorial Hospital, Taipei, Taiwan

**Keywords:** public health, aging, Daoyin, body constitution, sleep, autonomic nervous system, psychological wellbeing

## Abstract

**Introduction:**

This study assessed the potential of the Qi and Mind Harmonizing Method—a traditional Daoyin practice—as a community-based mind–body intervention to improve body constitution, cardiovascular function, sleep, and psychological wellbeing in older adults.

**Methods:**

An 8-week pre-post intervention trial was conducted at community centers. Daily practice was combined with weekly 60-min group sessions. Primary outcomes included changes in body constitution. Secondary outcomes included regional body constitution scores, brachial artery blood pressure, PSQI, HRV indicators, and BSRS-5.

**Results:**

After 8 weeks, 90 participants showed significant improvements in body constitution: Yang deficiency (−1.9, *p* = 0.002), Yin deficiency (−2.3, *p* < 0.001), and phlegm stasis (−2.2, *p* < 0.001). Improvements were observed in various body regions. Systolic pressure (−3.6 mmHg, p = 0.015), mean arterial pressure (−1.9 mmHg, *p* = 0.046), and pulse pressure (−2.4 mmHg, *p* = 0.037) decreased. Sleep quality improved (PSQI −1, *p* = 0.002). HRV analysis showed reduced LF, LF (%), and LF/HF ratio (*p* = 0.018–0.022), and increased HF (%) (*p* = 0.028). BSRS-5 scores improved from 3.9 to 3.2 (*p* = 0.009), indicating better psychological wellbeing.

**Discussion:**

The results may provide preliminary support for considering the integration of Daoyin into scalable public health approaches to healthy aging.

**Clinical trial registration:**

Funded by the Ministry of Health and Welfare, Taiwan, Republic of China; ClinicalTrials.gov number, NCT03640169. Registered on July 20, 2018.

## 1 Introduction

Since the World Health Organization’s first International Conference on Health Promotion in Ottawa in 1986, and through subsequent global health promotion conferences—including the 9th Global Conference in Shanghai (2016)—WHO has continued to refine and expand the principles of health promotion. These evolving frameworks emphasize not only community empowerment but also public participation (1, 2). Building on these foundations, the Ottawa Charter’s definition of health promotion as “the process of enabling people to increase control over, and to improve, their health” has remained a cornerstone in global public health. Moreover, WHO’s current strategy for Traditional, Complementary and Integrative Medicine (TCIM) (2025–2034) emphasizes integrating evidence-based and culturally respectful complementary practices into national health systems under appropriate regulation and community engagement (3–5). In Taiwan, these principles have been expanded to include traditional Chinese concepts of preventive medicine within public health planning (6), where mind–body practices such as Daoyin are recognized as culturally embedded approaches to health promotion (7–9).

Daoyin exercises originated in ancient China and have been practiced for thousands of years (10). The term “Dao” refers to the expulsion of turbid Qi and the intake of fresh air, while “Yin” involves physical movements to invigorate muscles and bones. This integrated practice of controlled breathing and coordinated body movement is designed to regulate internal Qi flow, promote meridian patency, and enhance musculoskeletal function (11). As a core modality in traditional Chinese medicine (TCM) and preventive care, Daoyin is recognized for its therapeutic and health-promoting potential. Common forms such as Qigong and Tai Chi fall under this umbrella and are widely adopted globally as complementary practices for strengthening physical resilience and managing chronic conditions, with growing empirical support for their efficacy (12–17). The practice of Daoyin is thus hypothesized to contribute to systemic physiological regulation and disease prevention.

Constitutional medicine, a cross-cultural field of study that emerged as a distinct discipline in the 19th century (18–21), continues to evolve through integration with contemporary health science (22–26). Broadly defined, constitution refers to relatively stable individual characteristics—shaped by both genetic predisposition and environmental influences—that affect health resilience, disease vulnerability, and physiological functioning. These characteristics include physical structure, energy regulation, and emotional tendencies. Among them, vital energy denotes the dynamic integration of energy and material substrates required for sustaining bodily function. This perspective aligns with the “narrow definition of constitution, “which focuses on systemic physiological regulation and serves as the conceptual framework for this study (27).

In Traditional Chinese Medicine (TCM), human life is conceptualized as an interplay between energy (Yang, representing physiological activity) and matter (Yin, the material substrate of the body). Optimal health is thought to depend on the dynamic balance and integration of these two forces. Vital energy, or constitution, serves as a key indicator of the body’s internal regulatory capacity and resilience. An imbalance or depletion of this vital energy may reflect transitions from health to sub-health or preclinical disease states, thereby signaling increased vulnerability to illness (28, 29). Maintaining a well-balanced vital energy is thus essential for sustaining physiological stability and preventing disease onset (30, 31). Yang deficiency” (Yang-Xu), “Yin deficiency” (Yin-Xu), and “Stasis” are TCM terms used to describe constitution, respectively indicating a persistent “lack of energy,” “lack of matter,” and “accumulation of pathological substances” owing to the obstruction in the movement of matter by energy (32). A Yang deficiency constitution may present with fatigue, cold intolerance, and loose or watery stool (33); a Yin deficiency constitution may present with oral ulcers, constipation, and hot flushes (34); and a Stasis constitution may present with chest tightness, easy bruising, and numbness of limbs (32). These three types of constitutions can appear individually or in combination. Conversely, if none of these three constitutions is present, it is considered a “balanced constitution.” Additionally, the distribution of energy and matter may be the same or different in different parts of the body, such as the head, chest, abdomen, and limbs (35).

Sleep quality is closely related to health. High-quality sleep enhances immune function, improves cognitive abilities, stabilizes mood, and maintains metabolic balance. Conversely, poor sleep quality can lead to decreased immunity, cognitive decline, psychological disorders, metabolic imbalances, and cardiovascular diseases (36). Blood pressure (BP) is an important indicator of cardiovascular health (37). Normal BP ensures proper blood supply to the organs and tissues, thereby supporting various bodily functions. However, high BP increases the risk of cardiovascular disease (38), stroke (39), kidney disease (40), and other health problems (41). Therefore, sleep quality and BP are key indicators of concern for health promotion policies. Increasing evidence suggests that autonomic nervous system function plays a critical role in regulating these factors, with heart rate variability (HRV) emerging as a key biomarker of autonomic balance (42). Policies promoting relaxation and stress resilience often target improved HRV, as lower LF/HF ratios and higher HF% are linked to better cardiovascular and mental health (43). Psychological distress, assessed by the Brief Symptom Rating Scale-5 (BSRS-5), is another crucial public health indicator, with higher scores associated with anxiety, depression, and sleep disturbances. These factors highlight the need for integrative strategies addressing both physiological and psychological wellbeing (44, 45).

We hypothesized that Daoyin regulates Yin and Yang through breathing and movement, enhancing autonomic stability, improving circulation, optimizing sleep, and strengthening psychological resilience. To test this, we conducted a real-world study to evaluate its effects on health indicators and public health.

## 2 Methods

### 2.1 Study participants

We conducted this trial at the Community Activity Centers in Taipei City, Taiwan, from July 10, 2017, to September 30, 2017. The Institutional Review Board approved the study protocol (Case #: CMUH106-REC1-082). Participants with significant injuries or illnesses, mental disorders, positive pregnancy tests, or those planning pregnancy during the study were excluded. Eligible participants were aged 20 years and older. All the participants provided written informed consent.

### 2.2 Study design

**Figure 1:**
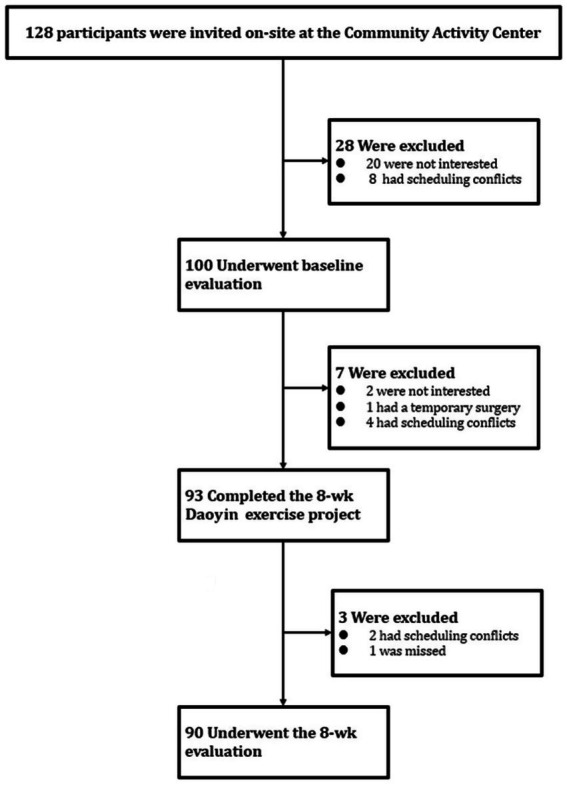
Invitation, baseline evaluation, and completion of 8-week evaluations.

This was a prospective, single-arm, pre–post intervention study conducted in a real-world community setting. No randomized control group was established, as the primary objective was to assess the feasibility and health promotion effects of the intervention under naturalistic conditions. This design was chosen to align with community-based health promotion practices and maximize participant accessibility. The research process is illustrated in Figure 1 and was conducted in accordance with the trial protocol.

### 2.3 Daoyin intervention

A Daoyin master with 15 years of teaching experience led the intervention, ensuring alignment with public health promotion strategies that emphasize preventive care, community engagement, and accessible wellness practices. The initial session introduced the fundamentals of Daoyin and the Qi and Mind Harmonizing Method, providing detailed explanations of key movements and principles (Appendix 1), followed by instructor-led practical training. The exercises were broken down into simple, repetitive components, focusing initially on breathing awareness and gentle body movements. This gradual layering allowed participants to gain familiarity without cognitive or physical overload. To support sustained participation and health literacy, participants received instructional handouts and videos for home practice and were encouraged to join weekly 60-min group sessions held at local community centers.

Each session reinforced the theoretical foundations, breath regulation, physical movements, and relaxation principles central to the intervention. After standardized warm-ups, participants engaged in supervised practice with real-time feedback to enhance precision and safety, which helped consolidate their practice. During the intervention period, participants were instructed to maintain regular self-directed home practice and avoid other structured exercise routines. The research team recommended at least 15 min of daily practice using provided materials and asked participants to log their adherence (Appendix 2), helping to strengthen habit formation and self-awareness. This pragmatic model reflects key public health values—accessibility, empowerment, and sustainability—and supports its integration into community-based health promotion initiatives.

### 2.4 Outcome measures

The primary outcome was the change in body constitution from baseline to the end of the 8-week intervention period. This study used the body constitution questionnaire (BCQ) (Appendix 3) consisting of three subscales. Some items were shared across the subscales, resulting in a total of 44. Each item was scored on a five-point Likert scale, with scores ranging from 1 (never happened) to 5 (always happened). The three subscales were as follows:

1. Yang-Xu: This subscale contained 19 items, with scores ranging from 19 to 95. A Yang-Xu constitution was indicated by a total score of 31 or above (27, 33, 46–48).
2. Yin-Xu: This subscale also contained 19 items, with scores ranging from 19 to 95. A Yin-Xu constitution was indicated by a total score of 30 or above (34, 47–50).
3. Stasis: This subscale included 16 items with scores ranging from 16 to 80. A Stasis constitution was indicated by a total score of 27 or above (32, 47, 48).

Higher scores indicate a stronger tendency towards the corresponding constitution type. For example, a higher Yang-Xu score indicates the presence of less energy. The Cronbach’s *α* values for the three subscales were approximately 0.85–0.88, and the overall Cronbach’s α value reached 0.90 (51).

Secondary outcomes during the 8-week intervention included the observed changes in energy and matter across different parts of the body, BP in the left brachial artery, heart rate variability (HRV), sleep quality, and psychological distress.

HRV was measured using the “Wegene” Handheld ECG Monitor (Certified by the Ministry of Health and Welfare, Medical Device Registration No. 004896). Measurements were conducted for 5 min while participants remained in a natural, awake state, avoiding movement and intentional breath control.

The study utilized the Pittsburgh sleep quality index (PSQI), developed by Buysse et al., as the measurement tool (52). Participants were asked to reflect on their sleep patterns over the past month, including the following seven components:

1. Subjective sleep quality — The participant’s satisfaction with their sleep quality.
2. Sleep latency — The amount of time it takes to fall asleep after getting into bed.
3. Sleep duration — The actual hours of sleep achieved each night on average.
4. Habitual sleep efficiency — The ratio of total sleep time to time spent in bed.
5. Sleep disturbances — The frequency and severity of disturbances during sleep.
6. Use of sleeping medication — The frequency of sleeping pill use per week.
7. Daytime dysfunction — The level of difficulty staying awake and maintaining enthusiasm during daily activities.

The global score, which is the sum of the scores of these seven components, ranged from 0 to 21. A global score greater than 5 indicates poor sleep quality, whereas a score of 5 or less indicates good sleep quality. In summary, a lower score indicates better sleep quality (52).

Psychological distress was assessed using the Brief Symptom Rating Scale (BSRS-5), a self-reported questionnaire completed by participants to evaluate their mental wellbeing (44, 45).

### 2.5 Statistical analysis

Descriptive statistics were used to analyze the baseline characteristics. Paired t-tests assessed changes in BCQ, PSQI, BP, HRV, and BSRS-5 values. Continuous variables are presented as means and standard deviations, whereas categorical variables are presented as frequencies and percentages. A *p*-value of < 0.05 was used as the threshold for statistical significance; *p*-values below 0.05 were considered significant, whereas p-values of 0.05 or greater were considered insignificant. All statistical analyses were performed using R software.

## 3 Results

**Figure 2:**
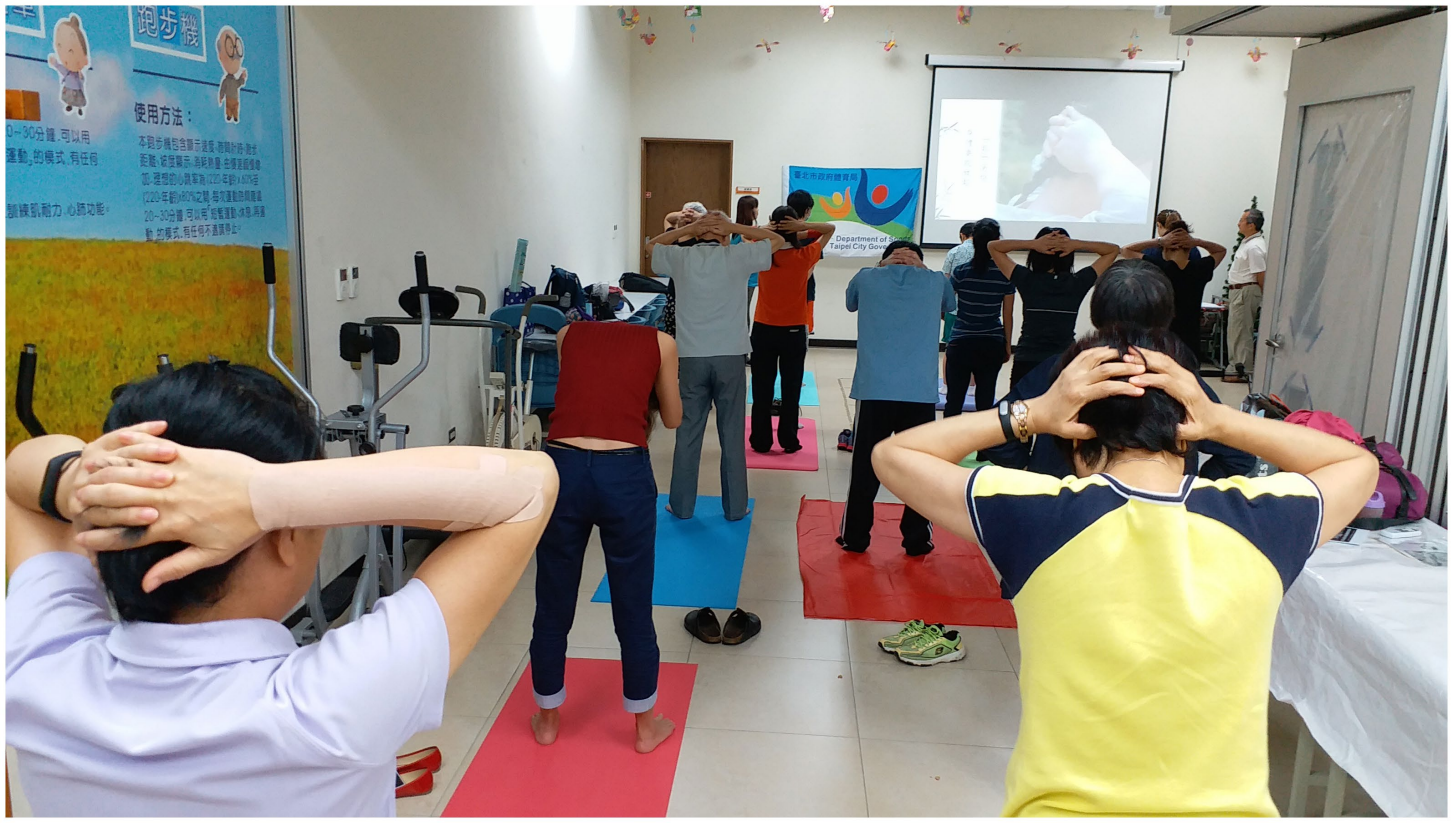
Community-based Daoyin instruction during the 8-week intervention. Participants followed guided group practice of the Qi and Mind Harmonizing Method at a local community center. Movements were synchronized with instructional video and real-time feedback from an instructor. No identifiable faces are shown.

Of the 128 individuals invited to participate in this community-based health promotion program, 100 completed the baseline assessment. During the 8-week Daoyin intervention (Figure 2 and Appendix 4), seven participants withdrew due to personal reasons, yielding a program adherence rate of 93%. Among the 93 who completed the intervention, three did not complete the post-intervention assessment. Ultimately, 90 participants completed both the pre- and post-assessments, resulting in a full program completion rate of 90% (Figure 1). These high rates of engagement and retention underscore the feasibility and acceptability of implementing Daoyin as a community-based health promotion strategy.

### 3.1 Baseline characteristics of the patients

The baseline characteristics of the 90 participants before the Daoyin intervention are described, including a mean age of 63.7 years, 66% women, and a mean body mass index of 22.8(Table 1).

**Table 1:** Baseline Characteristics of the Study Participants.*

Variable	Baseline
Female sex — no. of patients (%)	59 (66%)
Age—yr*	63.7 ± 11.2
Body weight (kg)*	58.6 ± 10.7
Body height (cm)*	160.0 ± 7.9
Body-mass index*^,^^#^	22.8 ± 2.9

* Plus–minus values are mean ± SD unless otherwise noted. # Body mass index is the weight in kilograms divided by the square of the height in meters.

### 3.2 Primary and secondary outcomes

Table 2 presents the changes in all outcomes from baseline to the conclusion of the 8-week intervention. In terms of primary outcomes, the BCQ scores for the Yang-Xu, Yin-Xu, and Stasis constitutions decreased significantly after 8 weeks. The specific reductions were as follows: Yang-Xu − 1.9 (95% CI, −3.19, −0.72; *p* = 0.002), Yin-Xu − 2.3 (95% CI, −3.5, −1.1; *p* < 0.001), and Stasis −2.2 (95% CI, −3.34, −1.12; p < 0.001). These scores were significantly lower than those recorded 8 weeks earlier (Table 2). Additionally, some participants transitioned from having a Yang-Xu constitution to no Yang-Xu constitution and from having a Yin-Xu constitution to no Yin-Xu constitution.

**Table 2:** Changes in BCQ score, blood pressure, HRV, PSQI and BSRS-5.^1^

Variable	Baseline	After 8-week intervention	*P-*value^2^
BCQ+(Yang-Xu)score^3^	31.2 ± 8.0	29.3 ± 7.2	0.002
Factor 1: Yang-Xu in the head	6.1 ± 2.0	5.7 ± 2.0	0.026
Factor 2: Yang-Xu in the chest	6.2 ± 2.0	5.6 ± 2.1	0.013
Factor 3: Yang-Xu in the four limbs	6.9 ± 2.4	6.3 ± 2.3	0.007
Factor 4: Yang-Xu in the abdominal cavity	6.0 ± 1.5	5.9 ± 1.5	0.334
Factor 5: Yang-Xu in the body surface	6.0 ± 2.5	5.8 ± 2.3	0.136
BCQ-(Yin-Xu)score^4^	31.0 ± 7.4	28.7 ± 6.7	<0.001
Factor 1: Yin-Xu in the head	10.0 ± 2.8	9.0 ± 2.7	<0.001
Factor 2: Yin-Xu in the four limbs	6.6 ± 2.3	5.9 ± 2.0	0.001
Factor 3: Yin-Xu in the gastrointestinal tract	6.5 ± 1.9	6.3 ± 2.0	0.293
Factor 4: Yin-Xu in the body surface	4.5 ± 1.5	4.3 ± 1.3	0.105
Factor 5: Yin-Xu in the abdominal cavity	3.4 ± 1.2	3.2 ± 1.3	0.097
BCQs(Stasis)score^5^	26.3 ± 7.4	24.1 ± 6.9	<0.001
Factor 1: Stasis in the trunk	8.1 ± 2.8	7.3 ± 2.6	0.002
Factor 2: Stasis in the body surface	7.8 ± 2.7	7.1 ± 2.3	0.001
Factor 3: Stasis in the head	5.5 ± 2.0	5.1 ± 2.1	0.013
Factor 4: Stasis in the gastrointestinal tract	4.9 ± 1.9	4.6 ± 1.6	0.117
Blood pressure^6^			
Systolic Pressure	131.2 ± 20.5	127.6 ± 19.1	0.015
Diastolic Pressure	76.7 ± 11.7	75.6 ± 10.9	0.208
Mean arterial pressure	94.9 ± 13.8	93.0 ± 12.5	0.046
Pulse Pressure	54.4 ± 13.62	52.0 ± 13.9	0.037
HRV(Heart Rate Variability)^7^			
LF	5.0 ± 1.3	4.7 ± 1.3	0.022*
HF	4.4 ± 1.4	4.3 ± 1.2	0.723
TP	6.5 ± 1.0	6.3 ± 1.1	0.164
LF(%)	60.4 ± 16.5	55.2 ± 19.5	0.022*
HF(%)	33.2 ± 14.7	37.8 ± 16.9	0.028*
LF/HF	0.7 ± 0.8	0.4 ± 0.9	0.018*
PSQI(Global score)^8^	8.0 ± 3.83	7.0 ± 3.3	0.002
Component 1: Subjective sleep quality	1.4 ± 0.8	1.2 ± 0.7	0.001
Component 2: Sleep latency	1.4 ± 1.0	1.2 ± 0.8	0.005
Component 3: Sleep duration	1.7 ± 0.8	1.8 ± 0.8	0.902
Component 4: Habitual sleep efficiency	1.2 ± 1.1	0.7 ± 1.1	<0.001
Component 5: Sleep disturbance	1.3 ± 0.5	1.2 ± 0.5	0.058
Component 6: Use of sleeping medication	0.5 ± 1.0	0.4 ± 0.9	0.114
Component 7: Daytime dysfunction	0.6 ± 0.7	0.5 ± 0.6	0.181
BSRS-5(5-item Brief Symptom Rating Scale)^9^	3.9 ± 3.3	3.2 ± 2.9	0.009**

^1^ All values are means with 95% confidence intervals. ^2^ p-values were calculated using repeated measures analysis of variance. A *p*-value < 0.05 indicates a significant difference, whereas a *p*-value ≥ 0.05 indicates no significant difference. ^3^ The BCQ + (Yang-Xu) score was used to assess the level of energy deficiency. Scores ranged from 5 to 95. The criterion for judging the Yang-Xu constitution was that the total score of these 19 questions should be greater than 31, with higher scores indicating more energy deficiency. ^4^ The BCQ- (Yin-Xu) score was used to assess the level of material deficiency. Scores ranged from 5 to 95. The criterion for judging the Yin-Xu constitution was that the total score of these 19 questions should be greater than 30, with higher scores indicating greater material deficiency. ^5^ The BCQs (Stasis) score was used to assess the level of material accumulation. The scores ranged from 5 to 80. The criterion for judging the stasis constitution was that the total score of these 16 questions should be greater than 27, with higher scores indicating more material accumulation. ^6^ Systolic and diastolic pressures were measured in the left brachial artery of the participants using an electronic sphygmomanometer. Mean arterial pressure was calculated using the formula: “systolic pressure * 1/3 + diastolic pressure * 2/3.” Pulse pressure was calculated using the formula: “systolic pressure – diastolic pressure.” ^7^ HRV (Heart rate variability) includes the following indicators. LF (low-frequency power): Reflects overall autonomic nervous system function. HF (High-Frequency Power): Represents parasympathetic nervous system activity. TP (Total Power): Measures overall heart rate variability. LF(%) = low-frequency power in normalized unit, an indicator representing the sympathetic function. HF(%): Normalized high-frequency power, associated with parasympathetic activity. LF/HF Ratio: Calculated as Ln(LF(ms^2^)/HF(ms^2^)), used to assess sympathetic activity. ^8^ Scores on the Pittsburgh sleep quality index (PSQI) ranged from 0 to 21, with higher scores indicating worse sleep quality. ^9^ BSRS-5 (5-item Brief Symptom Rating Scale) is a validated screening tool for psychological distress, assessing five core symptoms: anxiety, depression, hostility, interpersonal sensitivity, and sleep disturbance.

In terms of secondary outcomes, the scores for Yang-Xu, Yin-Xu, and Stasis showed changes across different body regions (Table 2). For Yang-Xu, the score decreased by 0.4 in the head (95% CI, −0.73, −0.05; *p* = 0.026), by 0.6 in the chest (95% CI, −0.93, −0.11; *p* = 0.013), by 0.6 in the four limbs (95% CI, −1.1, −0.18; *p* = 0.007), by 0.1 in the abdominal cavity (95% CI, −0.44, 0.15; *p* = 0.334), and by 0.2 on the body surface (95% CI, −0.44, 0.15; *p* = 0.136). For Yin-Xu, the score decreased by 1.0 in the head (95% CI, −1.37, −0.59; *p* < 0.001), by 0.7 in the four limbs (95% CI, −1.12, −0.28; *p* = 0.001), by 0.2 in the gastrointestinal tract (95% CI, −0.58, 0.18; *p* = 0.293), by 0.2 on the body surface (95% CI, −0.47, 0.04; *p* = 0.105), and by 0.2 in the abdominal cavity (95% CI, −0.46, 0.04; *p* = 0.097). For Stasis, the score decreased by 0.8 in the trunk (95% CI, −1.31, −0.31; *p* = 0.002), by 0.7 on the body surface (95% CI, −1.16, −0.31; p = 0.001), by 0.4 in the head (95% CI, −0.79, −0.09; *p* = 0.013), and by 0.3 in the gastrointestinal tract (95% CI, −0.55, 0.06; *p* = 0.117). These results indicate that, while the improvements in Yang-Xu, Yin-Xu, and Stasis varied across different body regions, the overall scores for these three constitutions decreased, suggesting that the Qi and mind harmonizing method had positive effects on improving these constitutions.

For other secondary outcomes, systolic pressure decreased by 3.6 mmHg (from 131.2 to 127.6; *p* = 0.015), diastolic pressure decreased by 1.1 mmHg (from 76.7 to 75.6; *p* = 0.208), mean arterial pressure decreased by 1.9 mmHg (from 94.9 to 93.0; *p* = 0.046), and pulse pressure decreased by 2.4 mmHg (from 54.4 to 52.0; *p* = 0.037). These results indicate significant reductions in systolic, mean arterial, and pulse pressures. In addition to blood pressure improvements, heart rate variability (HRV) showed significant changes. The low-frequency (LF) component decreased from 5.0 to 4.7 (*p* = 0.022), while the high-frequency (HF) component remained stable (4.4 to 4.3; *p* = 0.723). Total power (TP) slightly declined (6.5 to 6.3; *p* = 0.164). Notably, LF percentage dropped from 60.4 to 55.2% (p = 0.022), while HF percentage rose from 33.2 to 37.8% (*p* = 0.028), reducing the LF/HF ratio from 0.7 to 0.4 (*p* = 0.018). These results suggest the intervention enhanced parasympathetic activity and autonomic balance.

After 8 weeks of practicing the Qi and mind harmonizing method, the overall sleep quality of participants, as measured by changes in PSQI scores, improved significantly, with a reduction of 1 from 8 to 7 (*p* = 0.002). The changes in the scores of the seven components were as follows: subjective sleep quality decreased by 0.2 (from 1.4 to 1.2; *p* = 0.001), sleep latency decreased by 0.2 (from 1.4 to 1.2; *p* = 0.005), sleep duration increased by 0.1 (from 1.7 to 1.8; *p* = 0.902), habitual sleep efficiency decreased by 0.5 (from 1.2 to 0.7; *p* < 0.001), sleep disturbances decreased by 0.1 (from 1.3 to 1.2; *p* = 0.058), use of sleeping medication decreased by 0.1 (from 0.5 to 0.4; *p* = 0.114), and daytime dysfunction increased by 0.1 (from 0.6 to 0.7; *p* = 0.181). These results indicate that the participants were more satisfied with their sleep quality, fell asleep faster after going to bed, and had a higher proportion of sleep time in their total bedtime. Psychological distress, measured by the BSRS-5 scale, significantly decreased. The total score dropped from 3.9 to 3.2 (*p* = 0.009), indicating improved psychological wellbeing. These findings highlight the intervention’s potential as an effective holistic health approach.

## 4 Discussion

The results of this real-world, prospective, pre–post intervention study suggest that the Qi and Mind Harmonizing Method, implemented as a community-based health promotion exercise, significantly improved health parameters relevant to preventive public health strategies. After 8 weeks of consistent practice, participants showed improvements in body constitution imbalances related to energy deficiency (Yang-Xu), material deficiency (Yin-Xu), and pathological accumulation (Stasis). Several participants transitioned from Yang-Xu and Yin-Xu to balanced constitutions. Reductions in systolic blood pressure with stable mean arterial pressure indicate decreased cardiac workload without compromising organ perfusion—key for aging populations (53). HRV analysis further demonstrated enhanced parasympathetic activity and improved autonomic regulation. Notably, PSQI and BSRS-5 scores also improved, suggesting better sleep quality and reduced psychological distress—supporting the method’s role in holistic health promotion strategies.

These findings suggest that daily self-directed practice of the Qi and Mind Harmonizing Method may strengthen the body’s functional reserves—both in terms of vitality and physiological support—while improving internal regulation and reducing the buildup of harmful byproducts. Such improvements contribute to a more resilient physiological state than baseline (54–56). These outcomes were measured using the Body Constitution Questionnaire (BCQ), a validated tool with established reliability. In the context of constitution theory, enhancing an individual’s physiological foundation may reduce disease susceptibility and promote prevention (30, 31). This is especially relevant in aging societies, where chronic illness, functional decline, and disability are growing public health concerns (57–60). By focusing on older adults, this study provides preliminary evidence supporting the method’s feasibility as a health promotion approach that enhances baseline health status in aging populations. These findings suggest its potential to prevent or delay age-related diseases. Further large-scale and longitudinal studies are warranted to evaluate its public health value. Various types of Daoyin exercises exist, and this study employed the Qi and Mind Harmonizing Method due to its emphasis on mental relaxation, breath regulation, and joint activation. These features reflect Daoyin’s traditional focus on calming the mind and mobilizing the body through coordinated movement and breathing. The integration of mental focus, smooth respiration, and core muscle activation may enhance autonomic function and promote deeper circulation. These physiological effects may help optimize internal transport and address imbalances associated with low energy, limited physiological reserves, and stagnant metabolic byproducts. Future research could explore underlying mechanisms at the molecular level (61). Additionally, this study identified region-specific physiological responses (Table 2), suggesting that Daoyin may offer targeted benefits depending on body region. These findings support its potential application in precision public health (55).

Our study had some limitations. First, this research was conducted in a real-world community setting, where we promoted the Qi and mind harmonizing method and encouraged participants to continue daily practice independently after the 8-week study to cultivate health-promoting habits. As a result, no control group was established, and long-term effects were not evaluated. Second, in Chinese culture, Daoyin is known to induce physical and mental wellbeing, making placebo effects difficult to assess. This study did not further investigate placebo effects; however, we did not emphasize expected outcomes during the study. Instead, we helped participants focus on their practice and avoided disclosing specific symptoms that might be alleviated. Third, most participants were community residents who considered themselves healthy and had no serious illnesses, so this study did not assess sub-health populations. Lastly, we observed varying degrees of improvement in Yang-Xu, Yin-Xu, and Stasis across different body regions; due to limited sample size, further validation is needed. Despite these limitations, the findings offer valuable preliminary insights into the real-world applicability of mind–body interventions in public health.

This study provides valuable preliminary evidence supporting the feasibility and effectiveness of Daoyin as a community-based mind–body intervention for health promotion. To further address the study’s limitations and strengthen the evidence base, future research should include control groups, monitor constitution changes after practice cessation, and compare with groups receiving only health education. Documenting comorbidities and increasing sample size will enhance the generalizability and validity of results. These methodological refinements are essential to verify Daoyin’s role in modern health promotion. By improving study design and expanding target populations, future research can better define how traditional mind–body practices contribute to sustainable public health strategies, particularly in aging societies.

## 5 Conclusion

This study underscores the potential of the Qi and Mind Harmonizing Method as a scalable, community-based approach for national health promotion. An 8-week program significantly improved body constitution, cardiovascular health, sleep, and psychological wellbeing. These findings not only affirm the role of accessible, culturally rooted practices in supporting healthy aging but also provide real-world evidence to inform preventive public health strategies and integrative health promotion efforts.

## Data Availability

The original contributions presented in the study are included in the article/Supplementary material, further inquiries can be directed to the corresponding author.
