# Supplementary material for: A community-based Daoyin program for health promotion: effects of the Qi and mind harmonizing method on body constitution for the health of older adults
Source: Front Public Health. 2026 Jan 5;13:1644273. doi: 10.3389/fpubh.2025.1644273 (PMC12812638; doi:10.3389/fpubh.2025.1644273)
Supplement: Supplementary file 1 [file Supplementary_file_1.docx]

**Appendix 1:** Qi and Mind Harmonizing Method（https://www.youtube.com/watch?v=3r1s3zMiB7w&t=80s）

Daoyin has been practiced in China for over five millennia. It is an ancient approach to health preservation and disease treatment. Dao (guide) refers to exhaling turbid air and inhaling fresh air, whereas Yin (pull) involves moving the body to invigorate muscles and joints. This practice uses breathing techniques to harmonize internal energy, coupled with body movements to ensure smooth flow through the meridians and joints. This Qi and Mind Harmonizing Method is a health exercise suitable for all ages, and it uses simple movements to relax muscles, open the meridians, and enhance the function of sensory organs and internal organs. Its principle lies in using external physical movements to regulate Qi and blood flow, thereby achieving mental tranquility. This ensures the attainment of harmony within and outside the body to prevent diseases and extend lifespan. This Daoyin exercise is easy and effortless. Key points during practice include relaxation, tranquility, and naturalness—relax your body, calm your mind, and move gently and naturally. Let’s embark on a delightful Daoyin journey together!

1. Stand relaxed and upright.
2. Shift your weight to your left foot, then:
3. Tap the right foot and gently rotate the right ankle 10 times, keeping your body upright.
4. Repeat the same with your left ankle and rotate 10 times.
5. Return to a relaxed standing position; relax your shoulders, bend your elbows, and gently rotate your wrists 10 times.
6. After wrist rotations, relax your arms by your sides. Stand with feet shoulder-width apart, knees slightly bent. Slowly bend forward, keeping your back as straight as possible.
7. In the bent position, lightly press your palms on your knees and gently massage your knees from inside to outside 10 times.
8. Keep bending, support your knees with your palms, and gently turn your head to the left. Return to center, then gently turn to the right, repeating 10 times.
9. After neck rotations, maintain the bent position; supporting your knees with your palms, slowly exhale turbid air through the mouth, and slowly inhale fresh air through the nose, repeating 5 times. When exhaling, slightly contract your abdomen, and when inhaling, let it gently expand. Breathe smoothly and naturally, without forcing.
10. Still in the bent position; extend your arms and with your palms, gently Push downward 5 times. Focus on activating your spine and joints. Push to the left 5 times. Push to the right 5 times. Push forward 5 times. Push backward 5 times.
11. Return to a relaxed standing position; extend your arms and with your palms, gently push downward 5 times. Focus on activating your spine and joints. Push to the left 5 times. Push to the right 5 times. Push forward 5 times. Push backward 5 times. Push upward 5 times.
12. Next, relax your hands by your sides; open your mouth and gently tap your upper and lower teeth together 49 times, counting silently. Keep a slight smile on your face while tapping your teeth.
13. Then, bring your hands forward, palms together, with elbows relaxed and straight. Rub your palms against each other 49 times, counting silently.
14. After warming your palms, close your eyes and gently cover them with your palms, ensuring they are slightly concave to avoid pressing on the eyeballs. Massage around the eye sockets 25 times, counting silently.
15. Next, interlace your hands behind your head, slowly bend your neck forward, then return to the original position. Repeat this 10 times. Ensure slow and relaxed movements when bending the neck.
16. Then, place your hands over your ears, with palms covering the ear holes and fingers resting on the back of your head. Place your index fingers over your middle fingers and flick them off, tapping the back of your head 25 times. This technique is known as “Beating the Heavenly Drum” in ancient ear-care practices.
17. After the “Beating the Heavenly Drum” exercise, place your hands on your waist and relax. Slowly rotate your waist, drawing circles, 10 times clockwise and 10 times counterclockwise.
18. Then, return to an upright position, gently shake your body to relax, calm your mind, and place your hands on your abdomen. Breathe relaxed and naturally 10 times.
19. Inhale gently, allowing your abdomen to slightly expand, and exhale, letting it slightly contract. Continue breathing smoothly and naturally, without force, to achieve the effect of harmonizing Qi and the mind.
20. Finally, bring your feet together and lower your hands to conclude this practice of the Qi and Mind Harmonizing Method.
