# Supplementary material for: A community-based Daoyin program for health promotion: effects of the Qi and mind harmonizing method on body constitution for the health of older adults
Source: Front Public Health. 2026 Jan 5;13:1644273. doi: 10.3389/fpubh.2025.1644273 (PMC12812638; doi:10.3389/fpubh.2025.1644273)
Supplement: Supplementary file 2 [file Supplementary_file_2.pdf]

Appendix 2: Practice diary

2017.07

MON	TUE	WED	THU	FRI	SAT	SUN	Instructions for Filling Out
					1	2	Please check the box in the diary after practicing the Qi and Mind Harmonizing Method on that day.
3	4	5	6	7	8	9	
10	11	12	13	14	15	16	
17	18	19	20	21	22	23	
24	25	26	27	28	29	30	

Appendix 2: Practice diary

2017.08

MON	TUE	WED	THU	FRI	SAT	SUN	Instructions for Filling Out
31 July	1	2	3	4	5	6	Please check the box in the diary after practicing the Qi and Mind Harmonizing Method on that day.
7	8	9	10	11	12	13	
14	15	16	17	18	19	20	
21	22	23	24	25	26	27	
28	29	30	31				

2017.09

MON	TUE	WED	THU	FRI	SAT	SUN	Instructions for Filling Out
				1	2	3	Please check the box in the diary after practicing the Qi and Mind Harmonizing Method on that day.
4	5	6	7	8	9	10	
11	12	13	14	15	16	17	
18	19	20	21	22	23	24	
25	26	27	28	29	30		
