# Supplementary figures and images for: A community-based Daoyin program for health promotion: effects of the Qi and mind harmonizing method on body constitution for the health of older adults
Source: Front Public Health. 2026 Jan 5;13:1644273. doi: 10.3389/fpubh.2025.1644273 (PMC12812638; doi:10.3389/fpubh.2025.1644273)

**Appendix 3**: BCQ（Body Constitution Questionnaire）


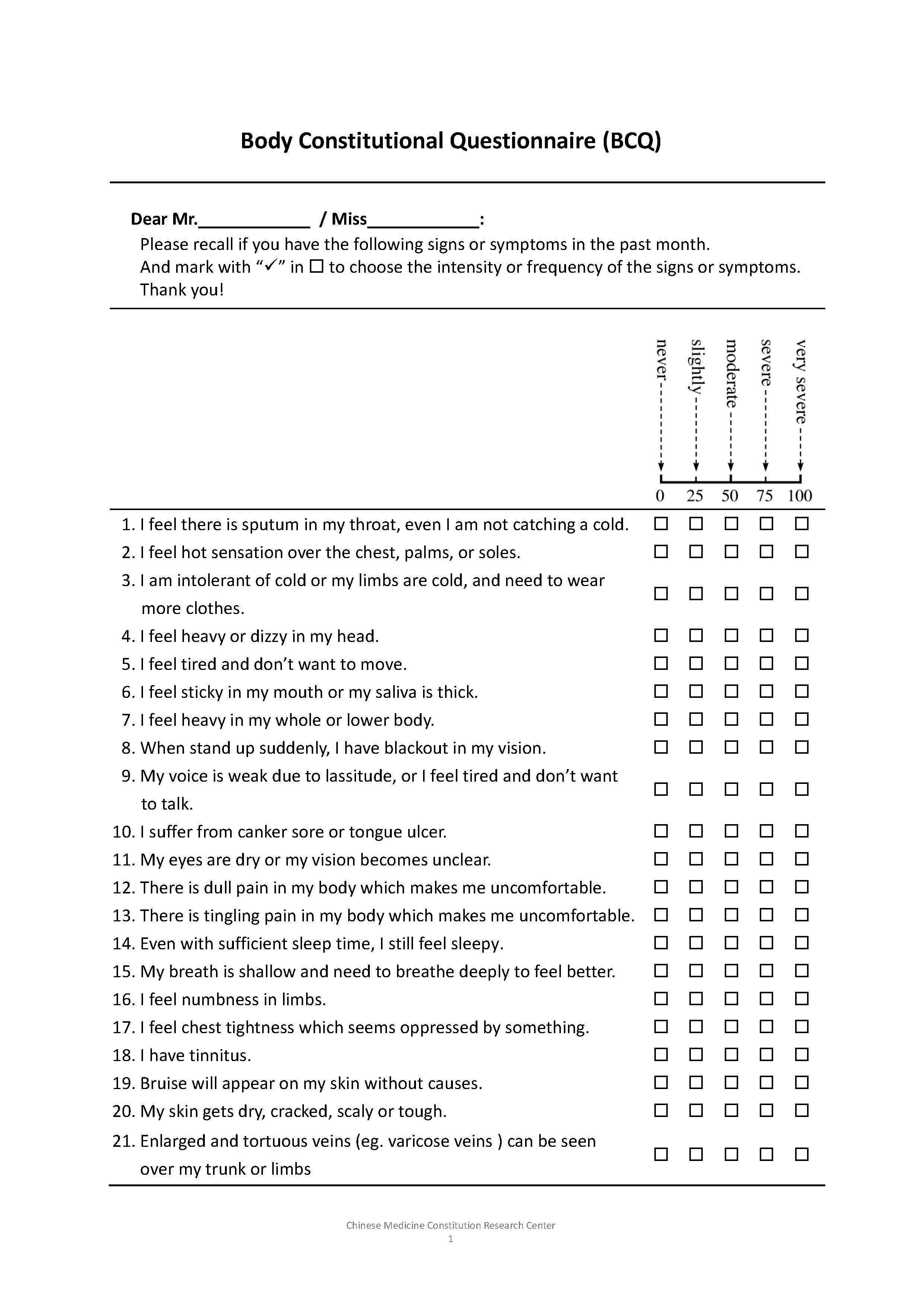


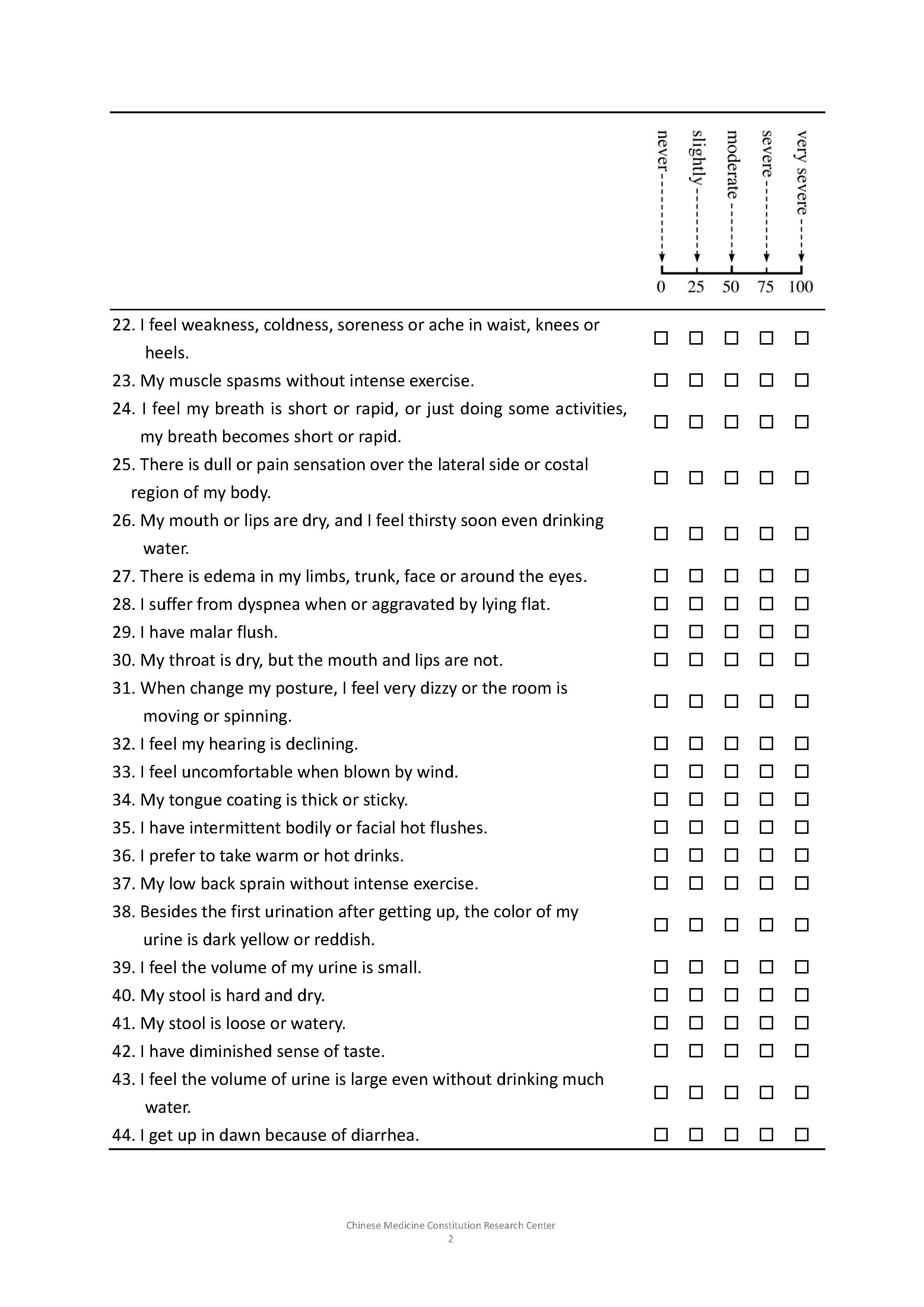

Supplement: Supplementary file 3 [file Supplementary_file_3.docx]
