# Supplementary material for: A community-based Daoyin program for health promotion: effects of the Qi and mind harmonizing method on body constitution for the health of older adults
Source: Front Public Health. 2026 Jan 5;13:1644273. doi: 10.3389/fpubh.2025.1644273 (PMC12812638; doi:10.3389/fpubh.2025.1644273)
Supplement: Supplementary file 4 [file Supplementary_file_4.docx]

**Appendix 4: Large-scale group practice of the Qi and Mind Harmonizing Method.**

**
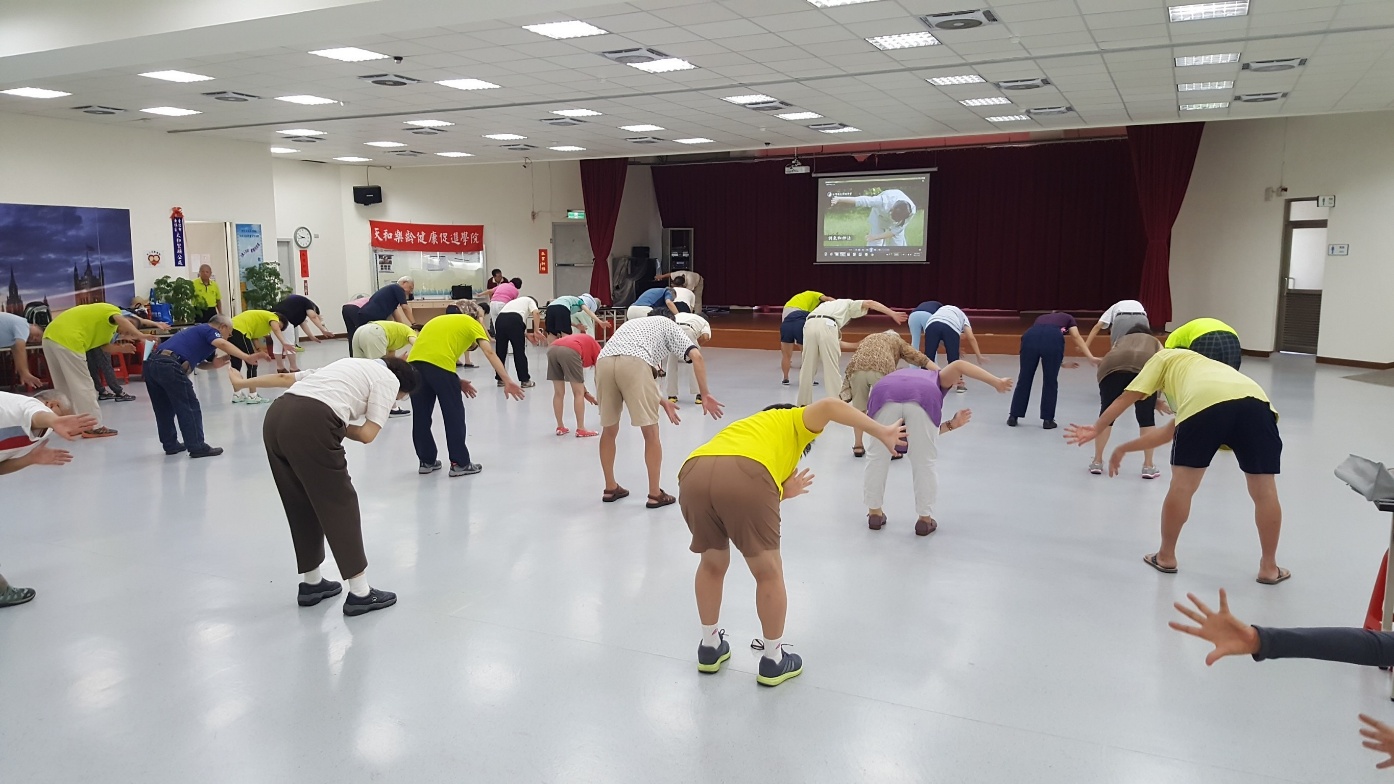
**

This photo captures a large-group Daoyin session held in a multipurpose community hall, demonstrating the scalability and feasibility of delivering mind–body interventions in public health settings. No identifiable faces are shown.
